# Photonic Crystal Structures with Tunable Structure Color as Colorimetric Sensors

**DOI:** 10.3390/s130404192

**Published:** 2013-03-28

**Authors:** Hui Wang, Ke-Qin Zhang

**Affiliations:** National Engineering Laboratory for Modern Silk, College of Textile and Clothing Engineering, Soochow University, Suzhou 215123, China; E-Mail: whui@suda.edu.cn

**Keywords:** photonic crystals, structure color, colorimetric sensors

## Abstract

Colorimetric sensing, which transduces environmental changes into visible color changes, provides a simple yet powerful detection mechanism that is well-suited to the development of low-cost and low-power sensors. A new approach in colorimetric sensing exploits the structural color of photonic crystals (PCs) to create environmentally-influenced color-changeable materials. PCs are composed of periodic dielectrics or metallo-dielectric nanostructures that affect the propagation of electromagnetic waves (EM) by defining the allowed and forbidden photonic bands. Simultaneously, an amazing variety of naturally occurring biological systems exhibit iridescent color due to the presence of PC structures throughout multi-dimensional space. In particular, some kinds of the structural colors in living organisms can be reversibly changed in reaction to external stimuli. Based on the lessons learned from natural photonic structures, some specific examples of PCs-based colorimetric sensors are presented in detail to demonstrate their unprecedented potential in practical applications, such as the detections of temperature, pH, ionic species, solvents, vapor, humidity, pressure and biomolecules. The combination of the nanofabrication technique, useful design methodologies inspired by biological systems and colorimetric sensing will lead to substantial developments in low-cost, miniaturized and widely deployable optical sensors.

## 1. Introduction

The publication of the pioneering work of Yablonovitch [1] and John [2] in 1987 may have started the intensive studies on photonic crystals (PCs) and sparked much of the modern interest in this field. PCs are materials that possess a periodic refractive index variance and have become a subject of high interest within the materials science community [3,4]. Due to the periodicity in dielectric materials, PC materials possess a photonic band gap (PBG), forbidding certain wavelengths of light located in the PBG from transmission through the material [5]. According to variations in the refractive index and period in space, PCs can be classified as one-dimensional (1D), two-dimensional (2D) and three-dimensional (3D). They have been intensively used in the area of optical fibers, photovoltaic devices, Bragg mirrors, displays, sensors and so on [3,4,6,7].

Recently, PCs have increasingly attracted the interest of researchers due to their unique structural color properties [7]. Photonic materials with vivid structural colors exist commonly in Nature, and are found in species of birds, butterflies, insects, marine life, and even flora [7–27]. Many organisms have the ability to tune their structural colors in response to surrounding environment for camouflage, warning about enemies or communication [7]. Inspired by these biological displays from Nature, PCs have been developed as chromotropic materials for colorimetric sensors. The sensors are created by combining materials that are responsive to external stimuli [28] such as solvents [29–33], vapors [34–38], temperature [39–46], ionic strength and pH [47–53], biomolecules [54–61], mechanical force [62–66] and so on. Colorimetric sensors are able to transduce environmental changes into visual color changes and are well-suited to the realization of low-cost and low-power sensors [34]. They provide an intuitively simple yet powerful detection mechanism based on the presence of PBGs that forbid the propagation of certain wavelengths of light in the visible range, negating the need for extra detectors by making environmental changes visible to the unaided eye. In order to satisfy the increasing number of requirements for actual application of colorimetric sensors, it is critical to develop smart artificial photonic materials with excellent sensitivity, response rate, durability and selectivity. The inspiration for the design and construction of photonic structures with vivid structural colors is extensively borrowed from nature and naturally occurring systems. Photonic structures capable of producing structural colors include 1D multilayer interference, 2D diffraction grating, and 2D/3D photonic crystals. Additionally, structural color is often dynamic, as PBG properties can be adjusted by external physical or chemical stimuli through manipulation of refractive index contrast and lattice constant in photonic crystal structures [28,67]. This review focuses on recent progresses in application of bio-inspired photonic materials with variable structural colors as colorimetric sensors.

## 2. Coherent Scattering of Light

**Figure 1.**
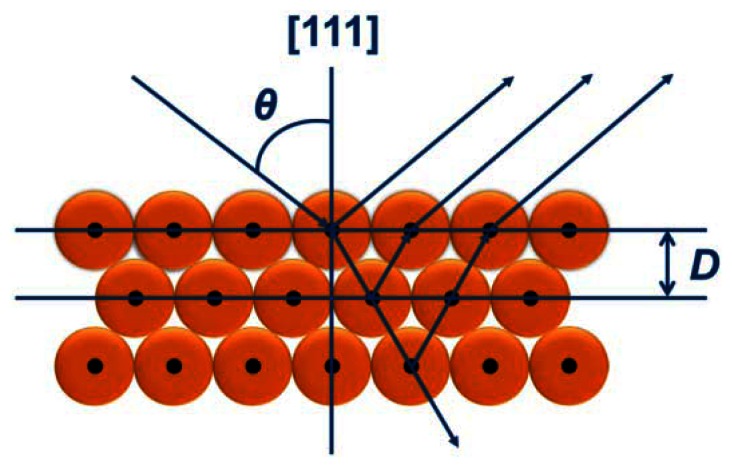
Incident light with a wavelength predicted by a modified Bragg-Snell equation (Equation (1)) undergoes diffraction when propagating through a PC. The wavelength of light that is coherently scattered is centered on *λ*, and can be estimated by the Equation (1) from the incident angle, *θ*, the effective refractive index of the PC, *n*_eff_, and the periodicity of the structure, *D*.

The colorful appearances of the PCs materials can be ascribed to interference and reflection, which can be described by Bragg's and Snell's laws [7,64] as shown in Figure 1. The law is given by:

(1) $$\lambda =2D\;{\left({n_{\text{eff}}}^{2}-\cos^{2}\theta \right)}^{1/2}$$

where *λ* is the wavelength of the reflected light, *n*_eff_ is the average refractive index of the constituent photonic materials, *D* is the distance of diffracting plane spacing, and *θ* is the Bragg angle of incidence of the light falling on the nanostructures. Based on the equation, there are several methods for tuning structural color, such as changing the diffracting plane spacing *D*, the average refractive index n_eff_, Bragg glancing angle *θ*, and changing the *n*_eff_ and *D* simultaneously. The dependence of *λ* on PCs material characteristics can be employed in the application of sensors. The use of photonic crystals as colorimetric sensors is the focus here. Colorimetric photonic-crystal sensors are based on structural colors tuned by external physical or chemical stimuli through the manipulation of refractive index and lattice constant.

## 3. Structure Colors from the Natural Photonic Crystals

### 3.1. Natural Photonic Nanostructures that Can Form Structural Colors

**Figure 2.**
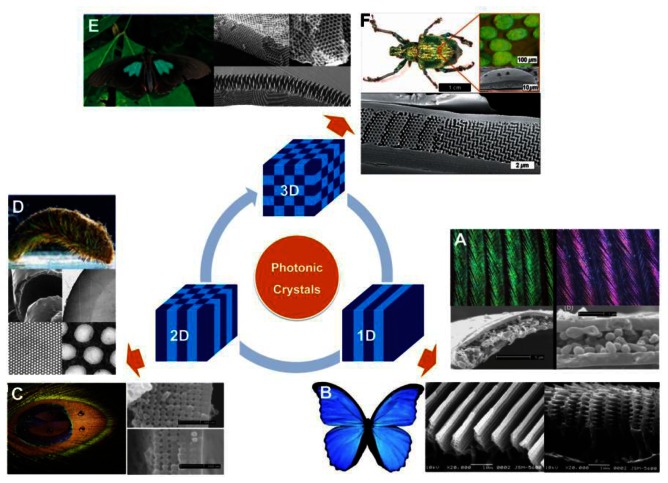
Typical naturally occurring photonic structures with various structural colors. (**A**) 1D periodicity in the form of multilayers existing in green and purple neck feathers of domestic pigeons [10]. (**B**) Some discrete 1D periodicity found in Morpho butterflies [8]. (**C**) 2D PC structure in the barbules of male peacocks with intricate, colorful eye patterns [14]. (**D**) 2D periodicity of cylindrical voids embedded in a high-refractive-index solid medium in iridescent setae from polychaete worms [9,15]. (**E**) 3D inverse opal structures appearing in the green color of *Parides sesostris* [7,9]. (**F**) 3D diamond-based photonic crystal structure in the beetle *L. augustus* [17].

Over millions years of evolution, living organisms have created an amazing variety of photonic structures to produce a colorful natural world. The structural colors generated by the photonic architecture in organisms have attracted a great amount of interest over time. These organisms have the ability to control the transportation of light using periodical photonic nanostructure units located on the surface of their bodies. In general, the bright structural colors of natural creatures play an important role in sexual attraction, social behavior and environmental camouflage [7]. According to variations of refractive index and period in space, natural PCs can be classified as 1D, 2D, and 3D frameworks, respectively, as shown in Figure 2.

Structural colors from 1D PCs often exist in insects, birds, fish, plant leaves, and berries with multilayered structures [8–13]. Such colors are mostly related to multilayer interference, although the structural color of pigeon neck feathers have been discovered to be caused only by the interference from one thin film [10]. Figure 2(A) shows the neck feathers of the domestic pigeon *Columba livia domestica*, with an iridescent green and purple color. The cross-sectional micrograph of the neck feather taken by the scanning electron microscope (SEM) shows green and purple barbules, both consist of an outer keratin cortex layer surrounding a medullary layer. There is an obvious difference in thickness between green and purple barbules. The interference in the top keratin cortex layer and total thickness of the layers decides the apparent color of the barbule. A more well-known example of naturally occurring multilayer interference is the brilliant blue color of *Morpho* butterflies' wings [8]. Electron microscope observation under high magnification clearly illustrates that a lamellar structure consisting of alternating layers of cuticle and air is present in each ridge (Figure 2(B)). The ridge-lamellar structure formed by discrete multilayers work as an element of quasi-multilayer interference, meaning the narrow width of height-varying ridges causes light diffraction without interference among neighboring ridges. The bright blue color is attributed to a significant difference in the refractive indices between cuticle (n = 1.56) and air (n = 1), with the layer thickness nearly fulfilling the conditions of ideal multilayer interference.

Compared to 1D photonic structures, 2D photonic structures in Nature provide richer color. Zi *et al.* reported the mechanism of color production in peacock feathers [14], finding that the differently colored barbules contain a 2D PC structure composed of melanin rods connected by keratin (Figure 2(C)). The nearly square lattice structures in the colored barbules differ in characteristics such as lattice constant (rod spacing) and number of periods (melanin rod layers) along the direction normal to the cortex surface. The tunable lattice parameters are the cause of the diverse coloration seen in the barbules. In addition, these 2D gratings exhibit self-cleaning capabilities due to the high fraction of the air trapped in the trough area between melanin rod arrays. Another type of 2D photonic structure is periodic long fibers found in the iridescent setae from polychaete worms (Figure 2(D)) [9]. A 2D hexagonal lattice of voids within the cross-section of each seta creates a natural pseudo-photonic crystal fiber along its full length. The high spatial periodicity of such lattice generates a partial PBG by which color is strongly Bragg-scattered in certain directions. As a consequence of the angle-dependent reflection, strong iridescence is observed laterally.

In nature, remarkable 3D PCs have the ability to produce partial PBGs, which can reflect bright colors over broad angles (Figure 2(E and F)) [9]. In principle, 3D periodicity potentially manipulates the flow of light in all directions. During early stages, scientists discovered 3D photonic structures in natural gemstones with iridescent colors [16]. A variation of the opal structure is the inverse opal structure, which was discovered in the wings of some species of butterflies (Figure 2(E)) [7]. Instead of close-packed colloidal crystals, the inverse opal structures in the butterflies are composed of lattices of hollow air-filled voids within a network of interconnecting cuticles. This photonic nanostructure appears to be a minor variation of the diamond-like tetrahedral structure, which offers excellent reflectivity over a broad angle range. In 2008, Bartl *et al.* discovered a diamond-based photonic crystal structure in the beetle *Lamprocyphus augustus* [ 17]. In contrast to the high dependence on angling shown by typical opal-like natural photonic structures with iridescence colors, this beetle displays a near angle-independent deep green structural color, as depicted in Figure 2(F). Observation via optical microscopy shows that such iridescence is due to individual leaf-shaped cuticle scales on the beetle's exoskeleton. The cross-sectional SEM imaging shows that each scale is composed of ABC stacked layers of hexagonally ordered arrays of holes and a staircase-like pattern in the surrounding cuticular matrix.

**Figure 3.**
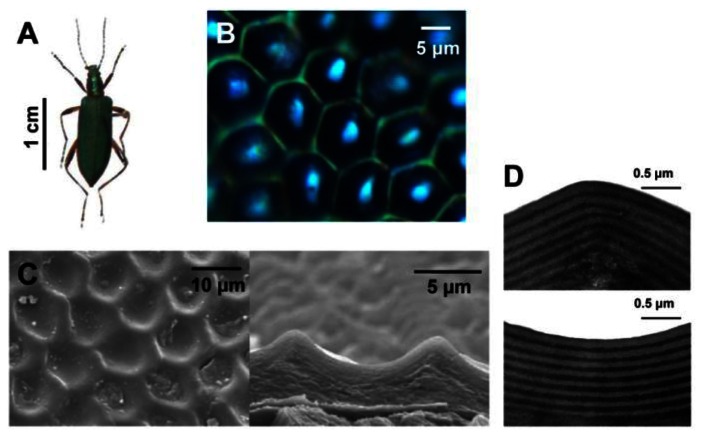
Naturally occurring composite multiscale photonic structures with variable structural colors. (**A**) The male *Chlorophila obscuripennis* beetle; its elytra displays an inconspicuous iridescent bluish green color. (**B**) Optical microscopic image of an elytron under 1,000× magnification. It is composed of a framework composed of green hexagonal veins and a cyan center. (**C**) SEM images of top view (left) and transverse cross-section (right) of the outer elytral surface. The elytral surface comprises of an array of hexagonal pits. (**D**) TEM transverse cross section of the ridge (top) and basin regions (bottom) of the outermost elytra [19].

Moreover, research of natural structural colors reveals that the functions of composite multiscale photonic structures and some amorphous structures are founded in nature [18–21]. Figure 3 displays the elytra of male beetles *Chlorophila obscuripennis*, which appears as an inconspicuous iridescent bluish green color when viewed with the naked eye [19]. However, from the top view under high magnification, the structural color displays a pattern consisting of a green color in the framework of hexagonal veins and a cyan color in the center. In contrast with the flat multilayer structural coloration in many other beetles, the elytra of *Chlorophila obscuripennis* possess a sculpted multilayer structure that combines 1D and 2D photonic structures, as reported from SEM and TEM observations. The average thickness of the bilayer (chitin and melanoprotein layer) in the sculpted multilayer is different in different regions, leading to different structural colorations. Moreover, the effective area for specular reflection is much smaller than that of a flat multilayer, leading to an inconspicuous structural bluish green color. Such sculpted multilayers were also found in butterflies, such as *Papilio palinurus* [18]. Their surface is comprised of a regular array of square pits. The sculpted multilayer was found to produce a yellow color at the basins and a blue color at the inclined sides, causing a mixed green color. These ingenious multiscale photonic structures of biological systems open exciting possibilities for the design of advanced optical materials.

### 3.2. Tunable Structural Colors of Natural Creatures

**Figure 4.**
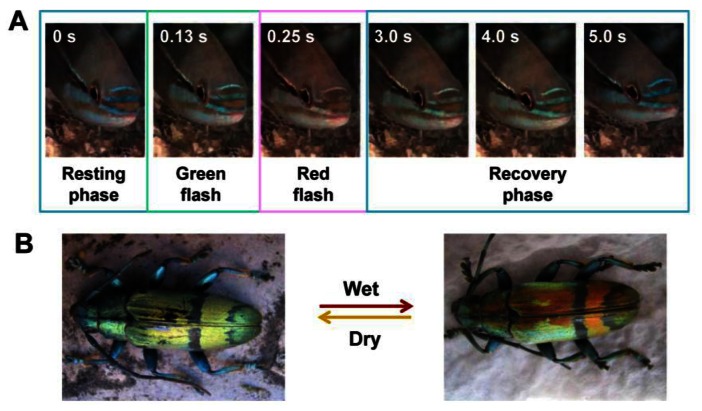
Natural creatures with tunable structural colors. (**A**) Structural color changes of the facial stripes in a paradise whiptail from blue to red through green and back to blue via yellow and green in 5 s [22]. (**B**) Structural color switches of the beetle *Tmesisternus isabellae* from gold in dry state to red in wet state [23].

In nature, some animals are able to reversibly switch their structural colors in response to their surrounding environments. This is for the purposes of disguising them to resist external attacks or fooling prey to trap as food, as shown in Figure 4. This phenomenon often exists in many fish and beetles, such as the paradise whiptail (*Pentapodus paradiseus*), neon tetra (*Paracheirodon innesi*), tortoise beetle (*Charidotella bicolor* (Fabricius)), Hercules beetle (*Dynastes hercules*), and others [7,22–25]. A typical system is based on the swell-caused spacing variation of 1D photonic multilayers, which leads to variable structural colors. For example, the paradise whiptail shows a cycle of color changes in its nose stripes (Figure 4(A)) [22]. Normally, due to the multilayer interference of light on the arranged iridophore plates in fish skin, structural color of blue is displayed during resting phase. Under different osmotic pressures, the fish rapidly changes coloration to red and restores its blue color, via expansion and shrinkage, respectively, of the spacing between platelets in hyperosmotic condition.

Similarly, the longhorn beetles *Tmesisternus isabellae* can alter their structural colors from golden, in dry state, to red, in a wet state (Figure 4(B)) [23]. Structural characterizations show a multilayer structure in the interior of the scales, consisting of two alternating layers existing in elytra of longhorn beetles, which produce the iridescent color. The small contact angle in the colored region indicates that the scales are hydrophilic, with the ability to facilitate water infiltration and absorption. The scale's structural color change to red in wet state is due to both the swelling of the multilayer period and water infiltration.

The structural color changes of biological organisms are based on changes in the refractive index and lattice constant, as influenced by external physical or chemical stimuli. The mechanism of naturally occurring tunable structural colors provides an inspiration for the design and fabrication of many types of artificial responsive photonic materials. Due to its unique properties, tunable structural color offers new opportunities for applications in colorimetric sensors and photonic devices.

## 4. Colorimetric Photonic-Crystal Sensors

As described above, many living creatures can reversibly change their structural color in response to external environmental stimuli. Inspired by the tunable structural colors of these natural creatures, great effort has been devoted to exploring the underlying mechanisms and developing techniques to mimic the tunable colors of photonic materials [67]. However, artificial photonic materials are much less smart than structures found in natural creatures.

To use photonic crystals as sensors, diffractions that fall into the visible range are usually preferred, as the optical output can be directly observed by the naked eye without the need of complicated and expensive apparatuses to read the signals. Moreover, considerations must be taken to ensure that the sensitivity, response rate, durability, and selectivity of the responsive system can meet the specific requirements of the sensor application. Generally, the photonic band gap can be reversibly changed in response to external physical or chemical stimuli. Relevant types of colorimetric sensors are discussed below.

### 4.1. Vapor and Solvent Sensors

**Figure 5.**
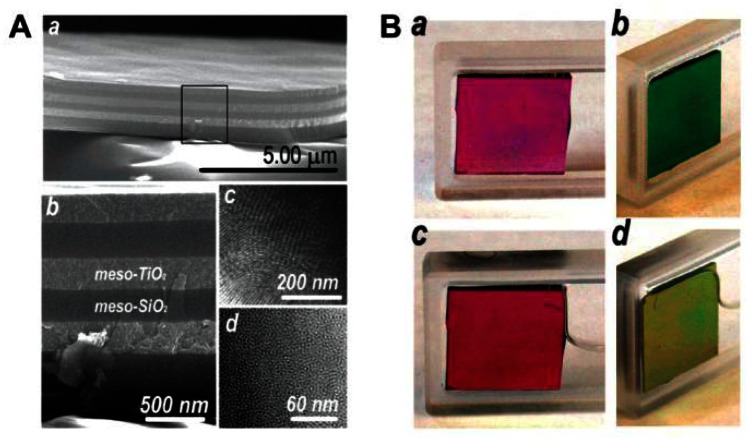
(**A**) SEM images of MBS composed of six alternating layers of mesoporous TiO_2_ and SiO_2_ (a and b), TEM images displaying the pore architecture of mesopropusTiO_2_ (c) and SiO_2_ (d). (**B**) Four-layer MBS in air (a and b) and in ethanol (c and d) observed from different viewing angles [29].

PC-based sensors analyze vapors and solvents by measuring the diffracting peak shift (color change) that often occurs during the change of effective refractive index and lattice spacing. Generally, it is difficult to tune the refractive index, as it is an intrinsic property of a bulk material. However, the average refractive index of a photonic structure can be changed by varying the components or tuning the refractive index discrepancy. As a result, the structural colors of the photonic materials change. Compared to 2D and 3D PCs, 1D PCs have an inherently simpler structure, which can be adopted to sense various chemical solvents and vapors when structured in multilayers [29–31]. 1D Bragg stacks composed of two kinds of alternating mesoporous layers have been studied to provide better resolution when two or more solvents with similar refractive indices need identification. This is due to the fact that both the composition and surface properties of each layer can be adjusted to enhance responses of the very subtle differences between various solvents. Ozin *et al.* reported mesoporous Bragg stacks (MBS) composed of spin-coated multilayer stacks of mesoporous TiO_2_ and mesoporous SiO_2_ (Figure 5(A)) [29]. The color can be reversibly altered by introducing or removing an analyte to or from MBS' pores (in ethanol, Figure 5(B)). The response of MBS depends on not only the refractive index of an analyte but also on other physical properties, such as hydrophilicity. Even in structures with very similar refractive indices, such as *n*-hexane (n = 1.375) and 2-propanol (n = 1.377), the response of MBS is distinct. The enhanced selectivity and sensitivity of MBS can be attributed to the change in composition ratio of mesoporous TiO_2_/SiO_2_, which possess different wettability. By increasing the relative portion of meso-SiO_2_, which is relatively more hydrophobic than meso- TiO_2_ in MBS, the response should have a larger spectral shift for the hydrophobic medium (alkane series), whereas the response to the series of alcohols decreased. However, the sensitivity for distinguishing among the alcohol series increased with the affinity to hydrophobic analytes increasing.

**Figure 6.**
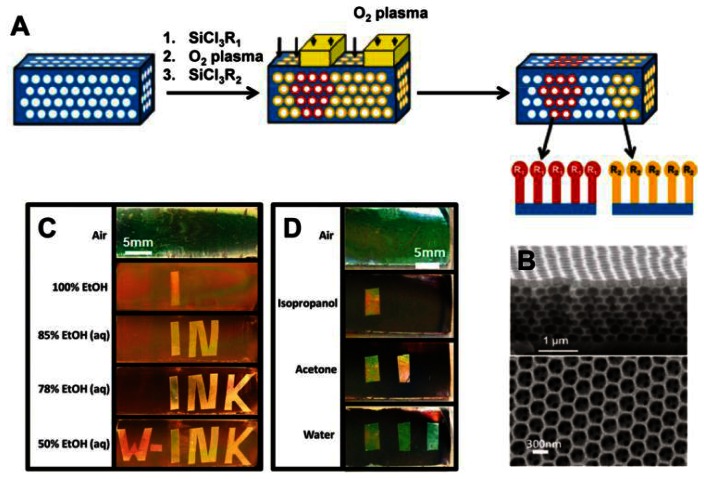
(**A**) Schematic of the chemical encoding procedure. The inner surfaces of IOFs are functionalized by repeated iterations of exposing the entire film to alkylchlorosilane vapors, followed by selective exposure to oxygen plasma. (**B**) SEM micrographs of cross section (top) and top view (bottom) on IOFs (**C**) Optical images of an IOF in which the word “W-INK” is encoded via the surface chemistry in an IOF. In different concentrations of ethanol, different words appear. (**D**) Optical images of a encoded film displaying distinct optical patterns when immersed in different solvents [33].

PCs forming close-packed 3D opals and inverse opals structure can often be employed for solvent sensors [32,33]. For such structures, the average refractive index can be adjusted by simply filling analyte into voids or pores, leading to a structural color change that corresponds to the wavelength shift of the stop-band. Because the inverse opals have a larger void percentage than opal structures, they lead to a more significant change in refractive index, and therefore higher sensitivity. Song *et al.* developed a carbon inverse opal to sense oil fabricated by using poly(styrene–methyl methacrylate–acrylic acid) (P(St–MMA–AA)) colloidal crystals as a template [32]. The different oils with different refractive indices were distinguishable via the unaided eye due to the defined color of the carbon inverse opal. The fast response time, durable oil-sensing stability and reversibility show that this carbon inverse opal shows promise for oil detection applications. In order to provide enough information to identify more solvents in one system, Burgess *et al.* presented a technique to fabricate chemically patterned SiO_2_ inverse opal films (IOFs) via multiple iterations of alkylchlorosilane exposure and masked oxygen plasma exposure (Figure 6(A and B)) [33]. When such patterned IOF soaked in a solvent, only regions with wettability above a specific threshold are expected to be infiltrated. Due to different refractive index discrepancy between wetted and nonwetted regions, a visible pattern forms (Figure 6(C and D)). Such functionalized regular geometry provides a high density of chemical information and allow many different patterns to form in different liquids with remarkable selectivity.

**Figure 7.**
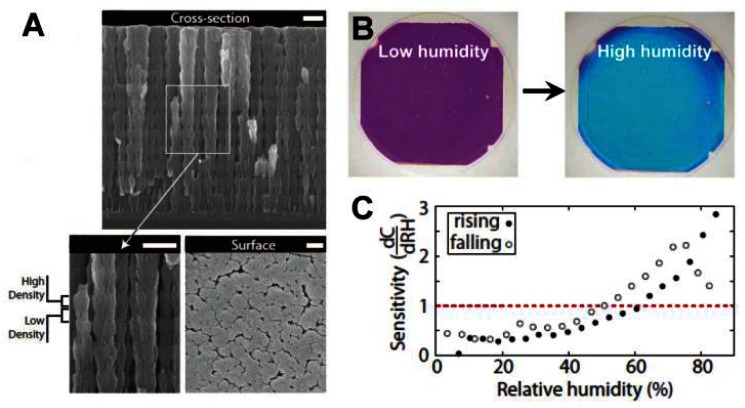
(**A**) Top view and cross-sectional view of the SEM images of as-prepared 1D TiO_2_ PCs films. The high and low-density regions of the structure are labeled. All the scale bars indicate 200 nm. (**B**) Colorful photographs of the sensor taken under the low and high humidity environments. (**C**) Sensitivity of device. Sensitivity values above the dashed red line indicate that a 1% RH change causes a detectable color change in the PC sensor [34].

PCs can be employed to sense not only solvents but also various chemical vapors. As the changes in the refractive index or the lattice spacing are determined by the filling ratio of the gaseous species, the vapor sensor actually measures the partial pressure of the vapor. A useful technical extension of such systems is the humidity sensor, which provides information of water vapor content in gaseous atmospheres [34–38]. For inorganic humidity sensors, structural color changes are often caused by changes in effective refractive index. Hawkeye *et al.* developed a mesoporous TiO_2_ PCs with high- and low-density structural layers constituting of high- and low-refractive index layers (Figure 7(A)) [34]. It is shown that the structural color changes of TiO_2_ PCs can be sensitively observed despite the fact that the relative humidity changes are smaller than 1%. The colorful response of the sensor lasts over hundreds of hours (Figure 7(B and C)). Hydrogel-based sensors generally induce a diffraction wavelength shift in response to humidity changes, owing to the volume change of polymer networks. Wang *et al.* developed a humidity sensor by infiltrating acrylamide (AAm) solution into a P(St–MMA–AA) photonic crystal template and subsequently photo-polymerizing [35]. The colors of such sensors could reversibly vary from transparent to violet, blue, cyan, green and red under various humidity conditions, covering the whole visible range. Furthermore, the color response showed exceptional stability under cyclic humidity experiments. Yang *et al.* reported an organic/inorganic hybrid 1D PCs consisting of alternating thin films of titania and poly(2-hydroxyethyl methacrylate-co-glycidyl methacrylate) (PHEMA-co-PGMA) by the simple, reproducible, and low-cost approach of spincoating [36]. Park *et al.* developed fast responsive polymeric humidity sensors from a series of self-assembled poly(styrenesulfonate-methylbutylene) (PStS-b-PMB) block copolymers with tailored hygroscopic properties [37]. Under different humidity, the PStS-b-PMB thin films displayed discernible reflective color changes covering almost entire visible light regions from violet (RH = 20%) to red (RH = 95%).

### 4.2. Temperature Sensors

Due to variation in materials, temperature sensors can be classified as both inorganic and organic sensors. Polymer-based temperature sensors detect temperature change based on fast optical-switching behavior caused by thermally induced reversible swelling and shrinking of the hydrogels. It generally have advantages in sensitivity, due to its greater volume change capacity. A typical system is composed of periodic materials based on a thermosensitive polymer gel of poly(N-isopropylacrylamide) (PNIPAM) [39–42]. PNIPAM with low toxicity can easily tune their response rate through controlling the microstructure of PNIPAM gel. Asher *et al.* developed a nanosecond photonic crystal switching material by using PNIPAM nanogel colloidal particles that self-assemble into crystalline colloidal arrays [39]. At a low temperature of 10 °C, these PNIPAM particles are highly swollen with a diameter of 350 nm. As the temperature increases, the particles shrink and expel water, with diameter decreasing to 125 nm at 40 °C. Smaller nanogels can efficiently control the diffraction shift caused by volume phase transitions on fast time scales. Takeoka *et al.* developed a technology to fabricate thermo-sensitive PNIPAM inverse opal gels by using relatively thick colloidal crystals as templates [40]. The polymer shrinks as temperature increases, causing the interparticle distance in the colloidal crystal to decrease and leading to diffraction shifting to shorter wavelengths. PNIPAM-based sensors generally have better sensitivity due to greater volume changes of polymer networks. However, such shrinkage is not suitable at high temperatures because of increases in the crack numbers and deformation of the ordered structure. Apart from using PNIPAM as thermo-sensitive materials, Thomas *et al.* presented thermally responsive self- assembled reflectors based on 1D photonic gels with periodic lamellar dielectric stack structure comprised of poly(styrene-b-isoprene) (PS-b-PI) block copolymer and cumene (a neutral solvent) [43]. Such copolymer photonic gels display a continuous blue shift when increasing temperatures from 30 °C to 140 °C in 10 °C increments. The most important factor contributing to the thermochromic effect in block copolymer gels is the variation of lamellar domain spacing due to temperature variation in degrees of segregation between the PS and PI blocks.

**Figure 8.**
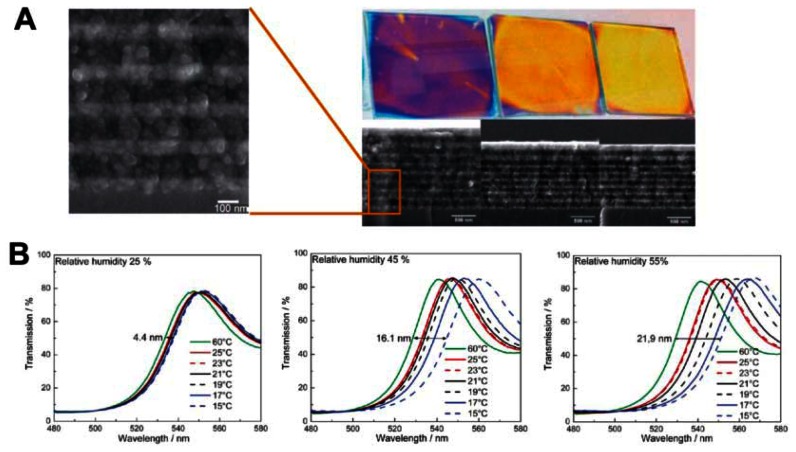
(**A**) Optical and SEM images showing the relationship between the color of the TiO_2_/SiO_2_ Bragg stacks and the thickness of the layers spin-coated at different spinning speeds. (**B**) Transmission spectra of the TiO_2_/SiO_2_ Bragg stacks demonstrating the blue shift in the temperature range between 15 °C and 60 °C and at various relative humidities (RH): 25%, 45%, and 55%. The magnitude of the shift increase with relative humidity increases [46].

Inorganic sensors detect temperature change based on the phase-transition-induced change of refractive index. They are good exceptional alternatives for polymer-based in broader application ranges due to its relatively high stability and wide refractive index range [44–46]. In order to obtain wider PBG by increasing the contrast in refractive index, sensor materials with higher refractive indices are necessary. It has recently become common for semiconductors with relatively high refractive indexes, such as TiO_2_ and SiO_2_, to be employed to construct various photonic structures [44,46]. Sato *et al.* reported colorimetric PC composite material by infiltration of nematic liquid crystals (LCs) into the voids of SiO_2_ inverse opal films [44]. As the refractive index of the LCs depend on the phases of LC molecules, phase changes affect structural color changes. LCs phases change from the nematic to isotropic as temperature increases, inducing the optical stop band to appear rapidly at the phase transition temperature, allowing its presence in the color of the film to be observed via the unaided eye. Such sensors can indicate the temperature change around their phase transition temperature. The switching rates of structural colors above and below the phase transition temperature are very fast. However, such sensors show a limited tuning range for diffraction wavelength. Lotsch *et al.* reported a thermally tunable and environmentally responsive optical filter derived from nanoparticle-based TiO_2_/SiO_2_ 1D PCs in the form of Bragg stacks, assembled by sequential spin-coating of stable colloidal suspensions of TiO_2_ and SiO_2_ nanoparticles (Figure 8(A)) [46]. Due to the porous nature of the multilayer, spectral shifts of different magnitude are observed at various relative humidities (Figure 8(B)). Notably, due to water adsorption-induced enlargement of the effective refractive index of the layers, the diffraction shift is significantly enhanced with increasing environment relative humidity, thus amplifying the thermal response and sensitivity of the Bragg stack.

### 4.3. Ion and pH Sensors

**Figure 9.**
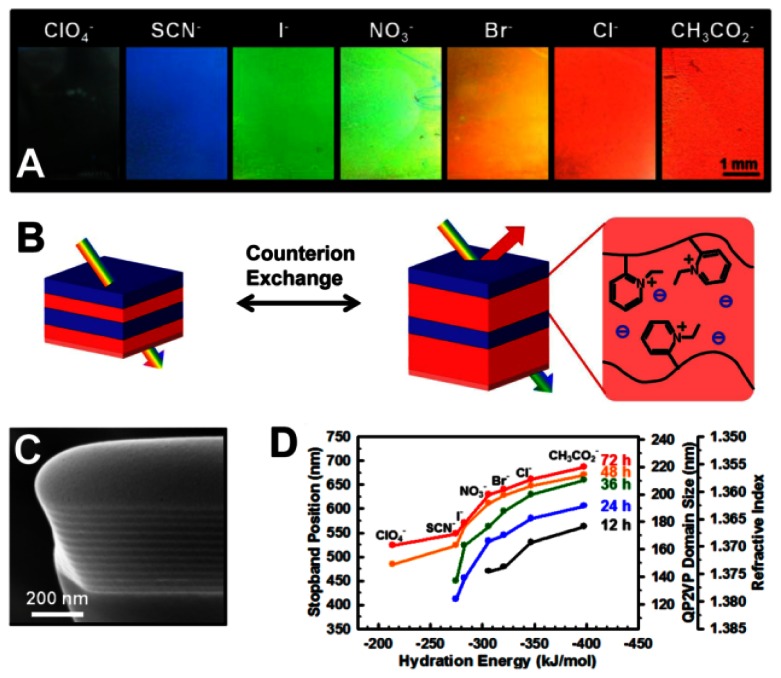
(**A**) Color change of the photonic gel films quaternized with direct exchange of counteranions. The color of the photonic gel films shifts from transparent to blue, green, and red as hydration energy of the counteranions increases. Reflective spectra and photographs of periodically ordered interconnecting porous gels. (**B**) Schematic representation of the mechanism for color change in the PS-b-QP2VP photonic lamellar gels via direct exchange of counterions. Depending on the hydration characteristics of the counteranions, the selective swelling in the block copolymer lamellar structures can be controlled, causing a change in optical properties. (**C**) Cross-sectional SEM image of a focused ion beam milled dry PS-b-QP2VP lamellar film. (**D**) Changes in the photonic stop band position, the QP2VP lamellar domain thickness, and refractive index of the tunable PS-b-QP2VP photonic gels as a function of the hydration energy of the counteranions [49].

Quantitative analysis of ions using PC sensors is accomplished by measuring the diffraction wavelength shift caused by changes in diffracting plane spacing. Ion sensors usually utilize the functional groups in hydrogels that are bound the corresponding ions, changing the geometrical structure of the PC hydrogel and thus causing diffraction wavelength shift [47–53]. Currently, many ion sensors are made from ion-sensitive PCs hydrogel via attaching the molecular-recognition group onto the polymer chains for selective binding of certain metal ions, such as Pb^2+^ and K^+^ [47,48]. Takeoka *et al.* prepared a gel with crown ether for capturing K^+^ selectively [48]. Such crown ethers swell and shrink reversibly, changing the lattice spacing of the PCs. The gel increases its volume with concentration of K^+^, caused by the positive internal osmotic pressure of the counterions. Red shifts of diffraction were observed by the naked eye as K^+^ concentration increased. However, the structural color was unaffected for sensing Na^+^. Thomas *et al.* presented a 1D periodic block copolymer photonic lamellar gels comprising the self-assembly of a hydrophobic block-hydrophilic polyelectrolyte block copolymer, quaternized polystyrene-poly(2-vinylpyridine) (PS-b-P2VP) with full-color tunability as a result of a direct exchange of counteranions (Figure 9) [49]. Due to the variances in hydration strength of the ions, the selective swelling of the block copolymer lamellar structure results in large tunability of the structural color of film from transparent to blue to red. The position of the PBG of the photonic gel films can be controlled by choosing hydration characteristics of the counteranions and degree of quaternization for quaternized P2VP microdomains.

**Figure 10.**
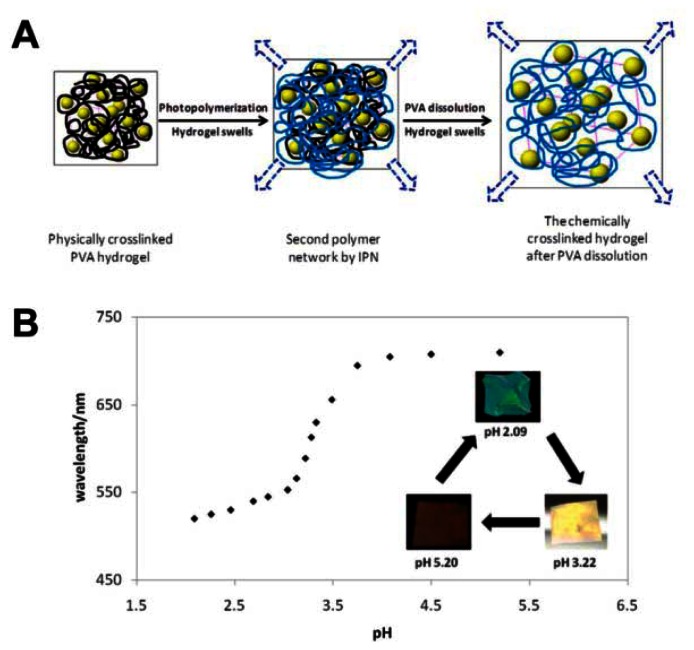
(**A**) Schematic illustration of the interpenetrating network (IPN) method for preparation of hydrogels with embedded crystalline colloidal arrays. (**B**) The pH titration curve for the acrylic acid composite hydrogel. Inserted images show the color changes of the hydrogel at different pH values [51].

As a special case for ion PCs, the pH-responsive PC detects the concentration of H^+^, an important parameter for many water-based reactions and analysis [50–53]. Braun *et al.* presented inverse opal hydrogel based on 2-hydroxyethyl methacrylate (HEMA) and acrylic acid (AA) copolymers, which exhibited pH-dependent shifts in optical diffraction [50]. The sensitivity of this pH sensor is tuned by varying the AA concentrations in hydrogel. The underlying mechanism of such pH sensors relate to the carboxyl groups on the polymer backbone that is ionized to carboxylate anions by increasing pH value. This results in the increase of Donnan osmotic pressure, swelling of the hydrogel and eventual shift of Bragg diffraction. Wang *et al.* reported a novel light diffracting hydrogel composite film consisting of acrylic acid/acrylamide copolymer with carboxylic group for H^+^ recognition and colloidal photonic crystals fabricated through a combined physical–chemical polymerization process (Figure 10(A)) [51]. The diffraction wavelength shift is so significant that the visible diffraction color change can be visually identified for the hydrogel (Figure 10(B)).

### 4.4. Biological Sensors

**Figure 11.**
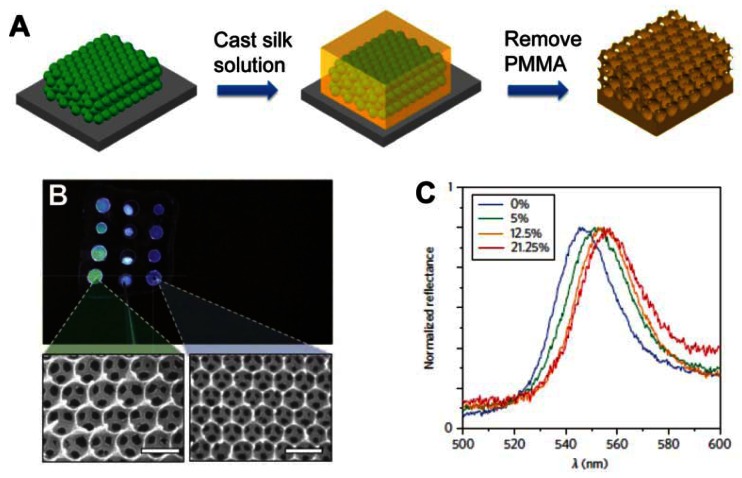
(**A**) Schematic illustration of fabrication steps for freestanding silk opal using a close-packed PMMA colloidal crystal as a template. (**B**) Photograph of a 2.5 × 1.5 cm freestanding silk film with patterned inverse opals. Corresponding SEM images show the green (left) and blue (right) silk opals. Scale bars, 500 nm. (**C**) Glucose refractive index sensing for the silk film with patterned inverse opals with lattice constants of 240 nm, showing reflectance spectra for different concentrations of glucose (0%, 5%, 12.5% and 21.25%) [55].

Recently, PC materials have been employed to design an optical biosensor for label-free bioassays when appropriate physical structures have been attached by recognition groups [54–61]. While in label-free detection, target molecules are unlabeled in their natural forms. The reactions of the target molecules and the PC sensor substrate should trigger physicochemical changes in PC materials, such as refractive index or diffracting plane spacing. These types of PC materials were often used as colorimetric glucose sensors [54–56]. Using the close-packed silica colloidal crystals template, Takeoka *et al.* created porous hydrogel PC film by using phenylboronic acid derivative immobilized hydrogel as materials for the framework [54]. Colorimetric response can be controlled by infiltration of structure with glucose. Using poly(methyl methacrylate) (PMMA) submicrometre spheres as templates, Omenetto *et al.* presented a free-standing silk film in the structure of an inverse opal (SIO film) with different lattice constants (Figure 11(A and B)) [55]. Once the film is exposed to glucose, the average refractive index of the PCs increases, leading to a red shift of the stop-band. Different concentrations of glucose solutions have different indices of refraction and result in different shifting of the PBG (Figure 11(C)).

**Figure 12.**
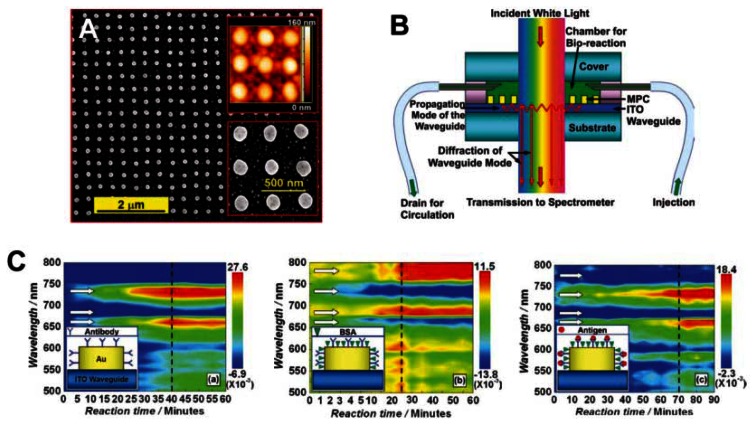
(**A**) SEM image of the 2D waveguided gold nanocylinders PC structures. Insets: AFM (top right) and enlarged SEM (bottom right) images. (**B**) Schematic illustration of the design and basic principles of the biosensor device based on the waveguided MPCs. (**C**) Immunoreaction-measurement results demonstrated by the sensor signal as a function of wavelength and reaction time in the antibody-binding process, BSA blocking process, and antigen-antibody reaction process. Insets: illustrations of the corresponding bioreactions [57].

Recently, sensors based on particle-plasmon resonance (PPR) have played an important role in biomedical engineering [56,57]. PPR sensors consisting of periodically arranged metallic nanostructures have attracted significant attention for their sensitivity to bimolecules and bioreactions. Zhang *et al.* reported waveguided, 2D, square lattices of gold nanocylinders with sensitivity to the HIV-1 virus. Detection was accomplished by using the specific interaction between the HIV-1 p24 antigen and the anti-p24 monoclonal antibody, an effective alternative to the RNA-detection approach (Figure 12) [57]. The reaction between the bimolecule and gold nanostructure, or among the bimolecules, induce a change in the environmental refractive index of the gold nanostructures, leading to a diffraction shift. The dynamic process of bioreaction taking place in the sensor chamber can be screened with a potentially high time resolution.

Currently, there is a rapidly expanding demand for methods to detect multiple biomolecules in a single assay for clinical diagnosis, gene expression, drug discovery, and so on. Most of these detections are based on molecular binding or recognition events. In order to distinguish different binding events in parallel, probe molecules are often immobilized on a substrate, with the coordinates of their positions encoded. Gu *et al.* developed bovine hemoglobin (Hb) imprinted photonic beads by template replication, used for label-free detection of bovine Hb at different concentrations without requiring immunological antibodies [58,59]. Biomolecules entering an imprinted nanovoid form abundant hydrogen bonds between oriented amide groups in the nanovoid and the polar surface residues of the biomolecules, resulting in significantly increased selective biomolecule-binding affinity. The imprinted photonic beads recognize the molecules through a gradual red shift in the Bragg diffraction, with an increase of the bovine Hb concentration. Remarkably, a trace amount of bovine Hb (1 ng·mL^−1^) was enough to cause a significant diffraction peak shift. For multiplex label-free bioassay, different spectral ranges encoded photonic beads imprinted with different proteins were mixed in a single tube containing the different analytes. The target proteins could be detected simultaneously.

### 4.5. Pressure Sensors

**Figure 13.**
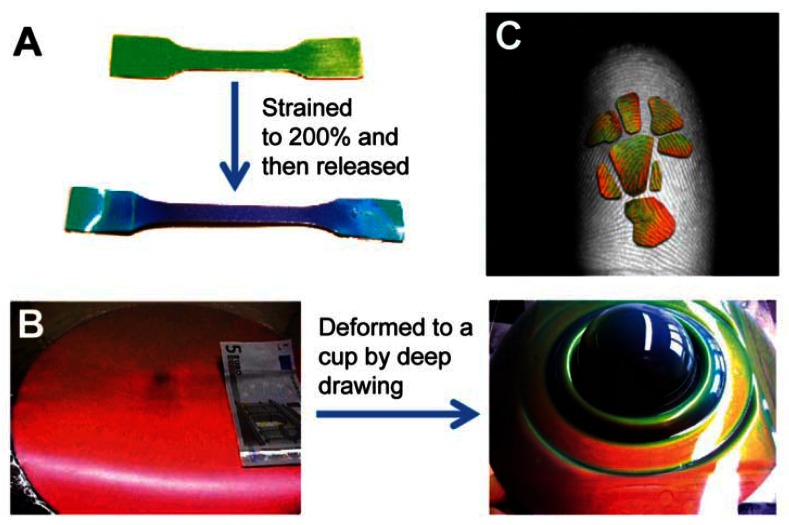
(**A**) Deformation of rubbery PC films obtained from polystyrene-poly(methyl methacrylate)-poly(ethyl acrylate) core-shell particles. Test bars before (green) and after (blue) 200% elongation of the PPC and release. (**B**) Elastomeric disk opal films prepared by compression molding before and after being deformed to a cup via deep drawing [63]. (**C**) A full-color fingerprint visualized using an elastic PC, overlayed onto a gray scale image of an index finger [64].

Pressure sensors prepared by PCs-based materials are based on the mechanical deformation of elastomeric PC composites, which induce their lattice constants to change and cause reflected color changes (Figure 13) [62–66]. Most of these pressure PC sensors are solid materials composed of colloidal crystals and soft polymeric frames; compression or stretch along one direction is normally accompanied by an expansion or contraction along perpendicular directions to maintain constant volume [62,63]. Hellmann *et al.* reported using a fast melt-flow technique to prepare a synthetic opal synthesized from beads with rigid polystyrene-poly(methyl methacrylate) cores and soft matrix-forming poly(ethyl acrylate) shell [63]. As the beads have a rigid core with an elastomeric shell, the films are able to tolerate considerable strain. Such deformation causes a strong shift of the reflected color from red to all colors across the rainbow spectrum (Figure 13(B)).

Elastic inverse opals have been used as mechanical stress tunable PCs. The color of highly porous architecture gradually shifts across the entire visible spectrum as light pressure is applied [64,65]. It was found that elastomeric inverse opal film stretched and released to enable the tuning of reflected light wavelengths [65]. In addition to stretching, pressure (compression) changes the layer spacing between the air voids, causing color to gradually shift across the entire visible spectrum when pressure is increased. Compared to non-porous solid materials, porous polymers can be compressed with minimal expansion in multiple directions. They can also be compressed perpendicular to the substrate. By compressing with a patterned elastomeric stamp, feature sizes down to 5 μm can be visualized using an elastomeric inverse opal film. Owing to the high sensitivity of pressure and striking color changes, such porous elastomeric films can be further developed into time and pressure-sensitive images to obtain a new generation of biometric recognition devices such as highly accurate color fingerprint readers (Figure 13(C)) [63]. The dramatic color changes can be directly observed when the material is pressed. This sensor can be reversibly used for many cycles, providing accurate and multichannel (pressure- and time-dependent) information for identification. This makes it an ideal security device for prevention of counterfeiting, helpful in distinguishing the imprint of a real finger from a rubber replica.

Apart from the application of PCs as colorimetric sensors for detecting the external stimuli, described above, electrically tunable PCs with full color display are also an important aspect colorimetric sensing. Recently, Ozin *et al.* reported a unique electrical PCs film comprised of an opal embedded in a matrix of a specialized redox-active polyferrocenylsilane (PFS) gel, an iron-based metallopolymer network [68]. Once a voltage is applied to the composite film, the metallopolymer gel swells and shrinks reversibly, leading to changes of the diffracting plane spacing, the structural color of the PCs materials was varied correspondingly. Varying the voltage, causes the color of the PCs materials to change, the color could shifted to any wavelength across the whole visible spectrum in several seconds. These electrically tunable PCs materials are well-suited to the realization of low-cost and low-power display units.

## 5. Conclusions/Outlook

In this review, we have summarized the recent developments on natural photonic materials with variable structural colors and applications of bio-inspired photonic materials as colorimetric sensors. It is noteworthy that various structural colors in living organisms generated by an enormous number of different photonic architectures have attracted a great amount of interest over time. Inspired by these natural creatures with tunable structural colors, great effort has been devoted to exploring the underlying mechanisms and the development of techniques to mimic natural photonic structures for applications as colorimetric sensors. However, artificial photonic materials are much less smart than the structures found in natural creatures. Quantitative analysis using photonic-crystal sensors is accomplished by measuring the diffraction wavelength shift caused by changes in effective refractive indexes and lattice constants through external physical and chemical stimuli. Recently, a great number of researches have been reported using PCs as solvent, vapor, temperature, pH, biomolecule, pressure sensors. Interestingly, a good PC is not necessarily an appropriate basis for a good colorimetric sensor, as the objectives of fabricating PCs and colorimetric sensors are not identical. To employ PCs as colorimetric sensors, it must be considered whether the sensitivity, response rate, durability, and selectivity of the responsive system can meet the specific requirements of the intended sensor application. Research of PCs-based colorimetric sensors is only beginning, and there are still numerous challenges. For example, the transition from laboratory to industrial practice requires large-scale manufacturing of these photonic structures in a highly efficient and reproducible manner. Major effort is still needed to further develop new approaches to meet the requirements for manufacturing. However, the effort is worthwhile, as PCs-based colorimetric sensors possess many advantages: the most prominent being the possibility of allowing to be observed directly without the aid of power sources and an expensive read-out system if reflected light wavelengths fall into the visible range. We firmly believe that the PCs-based colorimetric sensors have a bright and promising future.
